# Enhancement of the stability and cytotoxicity of berberine by liposomal nanocarriers for gastric cancer treatment and its application in gummy candy

**DOI:** 10.3389/fgstr.2024.1387343

**Published:** 2024-08-29

**Authors:** Narges Abolhasanzadeh, Gholamreza Dehghan, Soheil Abbaspour-Ravasjani

**Affiliations:** ^1^ Department of Biology, Faculty of Natural Sciences, University of Tabriz, Tabriz, Iran; ^2^ Drug Applied Research Center, Tabriz University of Medical Sciences, Tabriz, Iran

**Keywords:** berberine, nano-liposome, gummy candy, gastric cancer, solubility

## Abstract

**Introduction:**

Berberine (BER), an isoquinoline alkaloid derived from the plant Berberis Vulgaris, is traditionally used to treat different types of disorders, such as cancer. However, its therapeutic application is limited due to poor solubility and low bioavailability. So, the main objective of the present work was to synthesize BER-loaded liposomes to enhance the solubility of BER. BER-loaded liposomes were synthesized using the thin-film hydration method.

**Methods:**

The prepared liposomes were characterized for size, surface charge, in vitro release, and cytotoxicity. Then, the synthesized nano-liposomes were used to enrich gummy candies, and physicochemical properties such as water activity (aw), instrumental texture, and sensory perception of the products were tested.

**Results and Discussion:**

The cell viability assay was performed on MKN-45P gastric cancer cell lines, and the results revealed that BER-loaded liposome had better cytotoxicity on MKN-45P cells than free BER. The IC50 values were calculated to be 66.72 µg/mL and 52.58 µg/mL for free BER and BER-loaded liposomes, respectively. Flow cytometric analysis demonstrated a significant anticancer effect of BER-loaded liposomes compared to free BER. These findings indicate that encapsulating BER preserves its antioxidant activity and enhances its bioavailability.

## 1 Introduction

Cancer, the second cause of death globally, is a disorder described by the uncontrolled growth of unusual cells. Gastric cancer (GC), the third leading cause of cancer deaths, is one of the most common aggressive malignancies and has the fifth-highest incidence among cancers worldwide (1, 2). In 2018, approximately 782,685 deaths occurred due to GC worldwide (3). In recent years, the use of phytochemicals for the treatment of different disorders, such as various cancers, has gained considerable attention (4). Berberine (BER), as an isoquinoline alkaloid, is a natural product extracted from the herbal plant and belongs to the protoberberine group, which is present in various plant families such as Berberidaceae, Papaveraceae, and Ranunculaceae (5, 6). BER exists in the stem barks, rhizome, and roots of Berberis species and is mainly extracted from *Berberis vulgaris* (7, 8). Different pharmacological activities reported for BER are lipid-lowering and anti-inflammatory effects, management of insulin resistance, management of cardiovascular diseases, antioxidant activity, neuroprotection, inhibition of chondrocyte apoptosis and cartilage degeneration, anticancer, and antimicrobial properties (9–11). However, some limitations, including low water solubility and poor bioavailability, limited the application of these natural products. Thus, to overcome these problems, different delivery systems such as albumin-based nanoparticles, micelles, liposomes, and polymeric nanoparticles have been developed (12–15).

Among the types of nanoparticles used in drug delivery micelles are more stable than liposomes, have a smaller size range and the release of particles is more controllable by them. Polymersomes are similar to liposomes, except that they are composed of amphiphilic polymers, which are more stable and flexible than liposomes. Nanocarriers have been developed to transport some drugs that are unstable in the gastrointestinal tract. Therefore, these materials can improve the bioavailability and pharmacological properties of drugs. Liposomes, the most common nanocarriers, are widely used for drug delivery, but they have disadvantages that include fewer stables, low solubility, leakage, and fusion of encapsulated drug molecules, which can be overcome by specific strategies. These materials are phospholipid vesicles consisting of one or more concentric lipid bilayers enclosing discrete aqueous spaces (16, 17). The structural integrity of liposomes remains largely unchanged under gastric conditions for several reasons: gastric lipase lacks activity against phospholipids, thus they are not hydrolyzed in the stomach. Moreover, the liposomal membrane, being a lipid bilayer with a well-organized structure, contributes to its stability. Furthermore, cholesterol can form hydrogen bonds with phospholipids, thereby increasing membrane rigidity and further enhancing the structural stability of liposomes (18).

Thus, in this study, BER-loaded liposomes were generated by the thin-film hydration method and characterized using different analyses including particle size, zeta potential, polydispersity index (PDI) analysis, and scanning electron microscopy (SEM). Furthermore, the entrapment efficiency (EE) and *in vitro* drug release of BER-loaded liposomes were investigated. The potential use of BER-containing liposomes for the treatment of gastric cancer was evaluated against MKN-45. On the other hand, gummy candies containing free BER and BER-loaded liposome were produced, and the antioxidant activity and anticancer effect of the prepared gummy candies against gastric cancer MKN-45 cell lines were evaluated.

## 2 Materials and methods

### 2.1 Materials

BER, Phosphate buffered saline (PBS), 3-(4, 5-dimethylthiazol-2-yl)-2, 5-diphenyl tetrazolium bromide (MTT), cholesterol, and Twin 80 Sigma-Aldrich (St. Louis, MO, USA). Potassium persulfate, 1, 1-diphenyl-2-picrylhydrazyl (DPPH), and 2, 2′-azinobis 3-ethylbenzothiazoline-6-sulphonic acid (ABTS) diammonium salt were purchased from Merck Company (Darmstadt, Germany). RPMI 1640 medium, trypsin, and penicillin-streptomycin (pen/step) were prepared from Gibco BRL (Grand Island, NY, USA). Fetal bovine serum (FBS) and Annexin 5 were obtained from Biosera Company (Darmstadt, Germany) and Exbio, respectively. The MKN-45 and normal vascular endothelial cell lines (HUVEC) were provided by the Pasteur Institute of Iran’s cell bank. All chemicals used were of analytical grade and were used as received without further purification.

### 2.2 Methods

#### 2.2.1 Preparation of BER-loaded liposome

In this work, the thin-film hydration method was used for the preparation of liposome. For this purpose, 90 mg of lecithin and 10 mg of cholesterol, 10 mg tween 80, and 10mg BER (95%) were dissolved in 10 mL of ethanol. The prepared solution was evaporated to form a thin-film layer. The rotary evaporation continued until all traces of organic solvents were removed. The formed lipid film was hydrated using 10 mL of PBS to form the suspension of liposomes. Then the prepared solution was diluted with distilled water, homogenized for 15 minutes at 65°C using a Silent Crusher M (Heidolph, Germany), and sonicated by bath ultrasonic (Bandelin Sonorex Digitec, Germany) for 15 min. The suspension was stored at 2–8°C prior to characterization.

#### 2.2.2 Characterization studies

The morphology and internal structure of the prepared liposome were evaluated by using scanning electron microscopy (SEM; EM3200, KYKY Instruments, China). Also, the size and polydispersity index (PDI) of BER-loaded liposomes were studied using a DLS measuring system, Zetasizer Nano-ZS (Nano ZS3600, Malvern Instruments, UK). Furthermore, photon correlation spectroscopy (PCS) was used to determine the average zeta potential and size of BER-loaded liposomes using a Zetasizer Nano-ZS (Nano ZS3600, Malvern Instruments, UK).

#### 2.2.3 Determination of effective encapsulation rate

In order to determine the effective encapsulation rate (EE), 1 mL of BER-loaded liposome suspension was introduced into an Amicon filter (molecular weight cutoff of 100 kDa, Millipore, UK) and diluted with distilled water and then centrifuged for 10 min at 4000 rpm. The concentration of free BER (C_free_) in centrifugal fluid was determined spectrophotometrically by recording the absorbance of the prepared solution at a wavelength of 430 nm and using the following standard curve y=0.09643 x + 0.02630, which was constructed from a series of standard solutions of different concentrations of BER. EE and BER loading (DL) were calculated using the following equations (19):


 Y=0.09643*X+0.02630

Unload sample conc=Y= 0.09643 * 0.037+ 0.02630

Unload conc= 0.11

A unload sample: 0.037

$$EE\;\left(\%\right)=\frac{C_{T}-C_{AP}}{C_{T}}\times 100$$

$$DL\;\left(\%\right)=\frac{W_{DL}}{W_{NP}}\times 100$$

#### 2.2.4 Drug release from BER-loaded liposome

The dialysis method was used to study the BER release profile from synthesized liposomes. For this purpose, BER-loaded liposomes (2.0 mL) were dispersed in 100 ml of phosphate buffer solution (pH=7.4), poured into a dialysis bag, and placed into a shaking incubator at room temperature at 150 rpm. At various periods, 1.0 mL of buffered solution was collected, and the same volume of fresh phosphate buffer was added. The amount of released BER was measured spectrophotometrically by recording the absorbance of the samples at 345 nm, and the drug release curve was plotted.

#### 2.2.4 Formulation and preparation of gummy candies

To create gummy candies with optimal elasticity and flavor, adjustments were made to the ingredient proportions. The formulation of the gummy candy was slightly altered to enhance the bitter taste of BER. Ultimately, a gummy candy was prepared containing 30.17 g of liquid glucose, 250 mL of boiled water, two drops of peach essence, and 19.45 g of gelatin. The gummy candies were prepared using the following steps: The gelatin (19.45 g) was hydrated with distilled water and left to stand for 30 min. Also, glucose syrup (30.17 g) was dissolved in distilled water added to the hydrated gelatin, and mixed to homogenize the ingredients. The prepared mixture with the final volume of 250 mL was boiled for 30 min and then, flavor (peach) and yellow color were added to the composition. After cooling, we poured the pastilles into a silicone mold and put them in the refrigerator. After 4-5 hours, the pastilles were ready to use (19, 20). For the preparation of modified gummy candies, after mixing the gelatin and glucose syrup, 1ml BER (95%) and 1ml BER-loaded liposome were added and mixed well. Subsequently, flavor and yellow coloring were added, and gummy candies were prepared.

#### 2.2.5 Physicochemical properties

Sensory evaluation was performed with ten random consumers of the product, with the same number of females and males. The test consisted of a seven-point hedonic scale to reflect the preferences of consumers (1= very bad, 2= very bad, 3= relatively bad, 4= neither good nor bad, 5= fair, 6= very good, 7= very very good).

After preparing gummy candies, their physicochemical properties were studied. Texture profile analysis (TPA) was performed at 20˚C in a texturometer (LFRA-4500, Brookfield, USA). For this purpose, the prepared samples with a size of 15 mm ×15 mm were compressed twice at a rate of 1.0 mm.s^-1,^ and textural parameters including hardness, cohesion, springiness, gumminess, water activity, and chewiness were evaluated.

At 20°C, the water activity (aw) of the prepared gummy candies was measured using a Novasina Lab Master-aw neo water activity meter that had previously been calibrated with aqueous standards.

#### 2.2.6 Measurement of antioxidant activity by DPPH and ABTS methods

The antioxidant activity of prepared gummy candies containing free BER and BER-loaded liposome was investigated by DPPH and ABTS radical scavenging activity assays. For this purpose, a fixed concentration of gummy candies containing free BER and BER-loaded liposomes (gummy candies containing BER- loaded liposome been liquefied with distilled water and heat) were added to the 2.0 ml of ethanolic DPPH solution (0.1 mM). Then, the reduction of DPPH absorbance was recorded at 517 nm, and the DPPH radical scavenging activity (RSA%) of the samples was calculated using (21):


$$RSA\%=\frac{\left(A_{C}-A_{S}\right)}{A_{C}}\times 100$$

Where A_S_ and A_C_ are the absorbance of the prepared samples and the blank solution of DPPH (as a control) at 517 nm, respectively.

The ABTS radical scavenging activity was determined by dissolving desired amounts of potassium persulfate (2.45 mM) and ABTS (7.0 mM) in phosphate buffer (5.0 mN, pH=7.0) and incubating the prepared solution for 16 h in a dark room at 25 °C for the generation of green colored ABTS^+^. After centrifugation and filtration, the filtrate was diluted with phosphate buffer. Finally, 40 mg of BER-loaded liposomes was added to 4.0 ml of diluted ABTS, mixed vigorously, and kept in a dark room for 6 min at 25°C. The absorbance of the sample was monitored at 734 nm. The following equation was used for the calculation of the percentage of radical inhibition activity (%) (21):


$$\text{Scavenging of }734\text{ nm }=\;\frac{1-\text{Af}}{\text{Ao}}\times 100$$

Where, A_o_ and A_f_ correspond to the absorbance of the ABTS^+^ in the absence and presence of the samples, respectively.

#### 2.2.7 Cell culture

The gastric cancer cell line MKN-45 was cultured in RPMI-1640 medium supplemented with 2 g NaHCO_3,_ 1000 mL/L ddH_2_O, 10 mL/L penicillin-streptomycin antibiotics (1 MU/L penicillin and 10 g/L streptomycin), and 10 mL of heat-inactivated fetal bovine serum (FBS). Then the cultured cells were incubated in a humidified incubator with 95% air and 5% CO_2_ at 37°C.

##### 2.2.7.1 Analysis of cell viability by MTT assay

The MTT assay was used to investigate the cytotoxicity effects of free BER, and BER-loaded liposomes on the viability of the MKN-45 cell line. Briefly, the cells were seeded in 96-well plates at a density of 10,000 cells/well. By incubating for 24 h, the confluency of the seeded cells reached more than 70%. After removing the growth medium, the cultured cells were treated with different concentrations of free BER and BER-loaded liposomes (0-320 μM) (22) and incubated for 24 and 48 hours at 37°C. After 48 h of treatment, the culture medium was replaced with a new culture medium and the cells were washed twice with PBS. Then, the cells were treated with 50 µl of MTT solution (2 mg/mL) and incubated at 37°C. The culture media was withdrawn after 3 to 4 hours of incubation, and 200 µl of DMSO was added to each well to dissolve the purple blue formazan crystals. A microplate reader (Biotek, Winooski, VT) was used to record the absorbance of MTT color at 570 nm for each well, and the obtained data was analyzed by GraphPad Prism software version 8.

##### 2.2.7.2 Apoptosis assay

Apoptosis induced by BER and BER-loaded liposome was assessed using annexin V-FITC/PI staining for MKN-45 cells and the HUVEC cell line. For this purpose, MKN-45 cells were seeded in 6-well plates at a density of 5×10^5^ cells/well in 2 ml RPMI-1640 medium and then incubated at the standard condition for 24 h. The seeded cells were treated with the respective IC50 concentration of BER and BER-loaded liposome and incubated for 24h. After incubation, the seeded cells were harvested, washed with PBS twice, stained with annexin V-FITC/PI, and incubated for 15 min at 25°C in the dark. The data was analyzed with a flow cytometer (BD FACSCalibur), and the results were processed with FlowJo 7.6.1 software. Apoptosis-induced BER and BER-loaded liposome were also assessed using annexin V- FITC/PI staining. To assess apoptosis in the HUVEC cell line, the aforementioned conditions were employed to measure the apoptotic effects of liposome-encapsulated BER and free BER using Annexin V-FITC/PI staining.

### 2.3 Statistical analysis

All experiments were repeated three times. The obtained data from three independent experiments were presented as mean ± SD. The statistical analysis of the cytotoxic effect of BER and BER-loaded liposome in MKN-45 cells was performed by using GraphPad Prism v8.0.2 software.

## 3 Results and discussion

### 3.1 Evaluation of liposome, liposome size, the zeta potential of liposome suspension, encapsulation, and the BER-loaded liposomes

**Figure 1:**
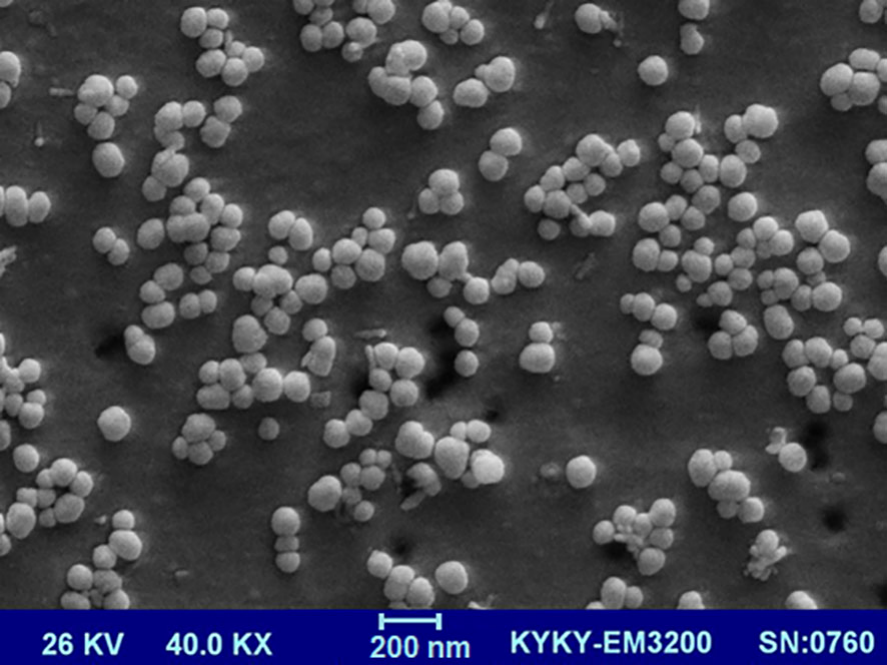
SEM image of BER-loaded liposome with 40.0KX magnification.

The morphology of the fabricated nano-liposome was evaluated using SEM analysis. **Figure 1** displays an SEM image of the synthesized nano-liposome. This figure shows the spherical vesicles and closed-continuous structures of the nanoliposome (23).

**Figure 2:**
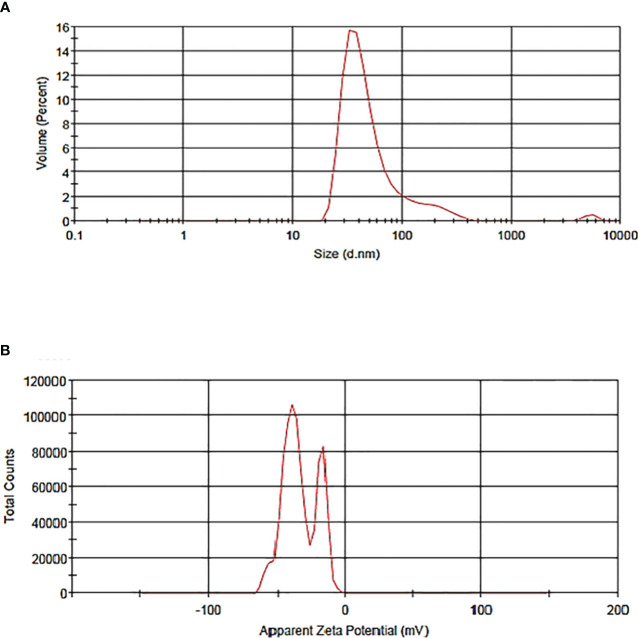
**(A)** The particle size distribution of liposome without BER, **(B)** zeta potential of liposome without BER, generated by the thin-film hydration technique.

**Figure 3:**
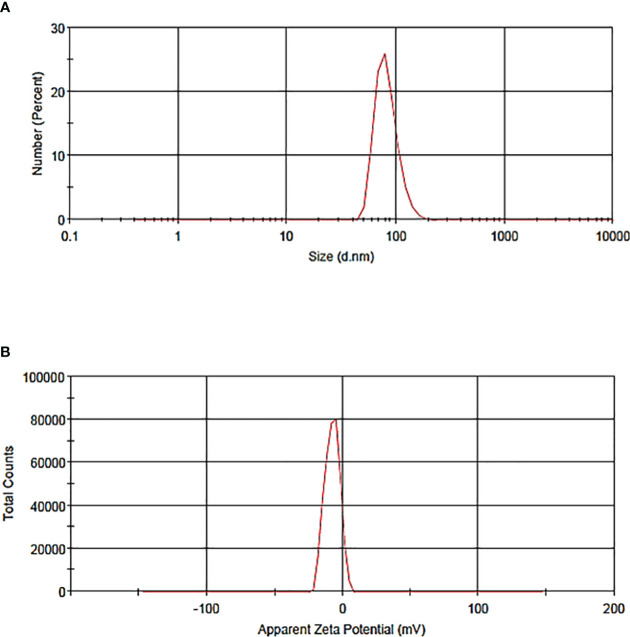
The particle **(A)** size distribution of BER-loaded liposome, **(B)** zeta potential of BER-loaded liposome generated by the thin-film hydration technique.

Vesicle size determination is an essential parameter for the application of liposomes, which guides the behavior of nanomaterials, stability, release profiles, biological distribution, and cellular uptake. The particle size distribution and zeta potential of the liposome without BER were 58.47 nm and -33.3 mV, respectively (**Figure 2**). The particle size distribution and zeta potential of the BER-loaded liposome were measured at 83.26nm and -7.58 mV, respectively (**Figure 3**). BER adheres to the surface of liposomes due to its positively charged amine group. Additionally, the three carboxylic groups in BER impart a negative charge to the liposome, enhancing the stability and dispersion of liposomal nanocarriers. By comparing the zeta potential of BER-loaded liposomes with liposomes without BER, we observed that after loading BER, the surface charge of liposomes remains negative (**Figure 3**).

PDI is used to describe the heterogeneity of a sample based on size, which ranges from 0.0 (for a uniform sample) to 1.0 (for a non-uniform sample with multiple particle sizes). PDI values greater than 0.7 show a very broad size distribution of particles (24). Therefore, these samples are not suitable for further studies. The calculated PDI value for BER-loaded liposome was 0.297, which is a suitable and acceptable value. These results indicate that liposomes maintain their homogeneous average sizes even after BER loading. The calculated PDI value for liposomes without BER was 0.458.

The effective EE is 99.9978% according to the calculations, which shows that 99% of BER is loaded in the liposome. Also, drug loading (DL) is 4.999%, so only 4.9% of the weight of liposomes consists of BER. Liposomes are distinctive biomolecules capable of encapsulating both hydrophilic and hydrophobic molecules for delivery into biological systems. Poorly water-soluble BER was observed to accumulate within the bilayered phospholipid membrane of liposomes prepared using the thin-film hydration method (25). The high DL and encapsulation efficiency (EE) are attributed to the ionic adsorption of BER on the inner and outer surfaces of the liposomes.


Y = 0.09643 * X + 0.02630

Unload sample conc =Y = 0.09643 * 0.037 + 0.02630

Unload conc= 0.11

A unload sample: 0.037

$$EE\;\left(\%\right)=\frac{C_{T}-C_{AP}}{C_{T}}\times 100$$

$$EE\;\left(\%\right)=\frac{5000-0.11}{5000}\times 100\;=\;99.9978\%$$

$$DL\;\left(\%\right)=\frac{W_{DL}}{W_{NP}}\times 100$$

$$DL\;\left(\%\right)=\frac{4.999}{100}\times 100\;=\;4.999\%$$

### 3.2 *In-vitro* drug release from produced nano-liposome

**Figure 4:**
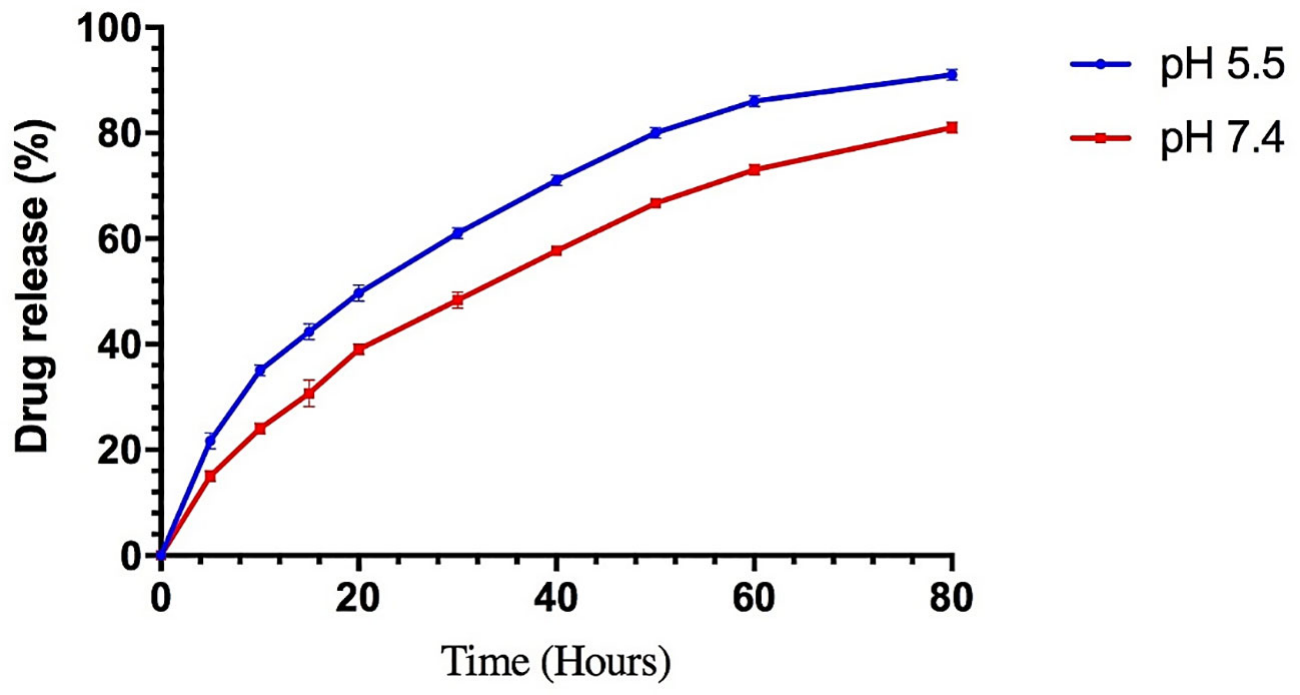
The *in vitro* BER release of the liposomes was prepared by the thin-film hydration method at pH 5.

For 80 hours, 2ml of the BER-loaded liposome release profile was monitored in PBS (pH 7.4 and 5.5) at 37°C. As shown in **Figure 4**, the release rate of BER from the liposomes demonstrated an initial burst release and the total amount of BER released from the liposomes was 81% (at pH 7.4) and 91% (at pH 5.5). Nanocarriers have been developed to transport drugs that are unstable in the gastrointestinal tract, enhancing their bioavailability and pharmacological properties. Liposomes, the most common nanocarriers, are widely used for drug delivery. However, they have disadvantages such as limited stability, low solubility, and the potential for leakage and fusion of encapsulated drug molecules. These issues can be addressed through specific strategies (16, 17). One of the key components of gummy candy is gelatin. Gelatin is a polyampholyte, possessing both positive and negative charges, which makes it an amphiphilic molecule with hydrophilic and hydrophobic groups. This characteristic enables gelatin to interact with oppositely charged biopolymers and adsorb at the oil-water interface, making it a versatile encapsulating agent for producing various nanocarriers (26). The prepared liposomes released BER in an acidic medium slower than in a neutral medium. It is evident that due to the unilamellar structure, liposomes are disrupted faster by acid (27). Recent studies on the use of gelatin as a nanocarrier have shown that it performs well at a pH of 5-7. In gummy candies, gelatin allows BER-loaded liposome to withstand the acidic conditions of the stomach and interact with the cancer cell membrane. This facilitates the effective release of BER (28, 29).

### 3.3 Analysis of the physicochemical properties of the designed gummy candy

**Figure 5:**
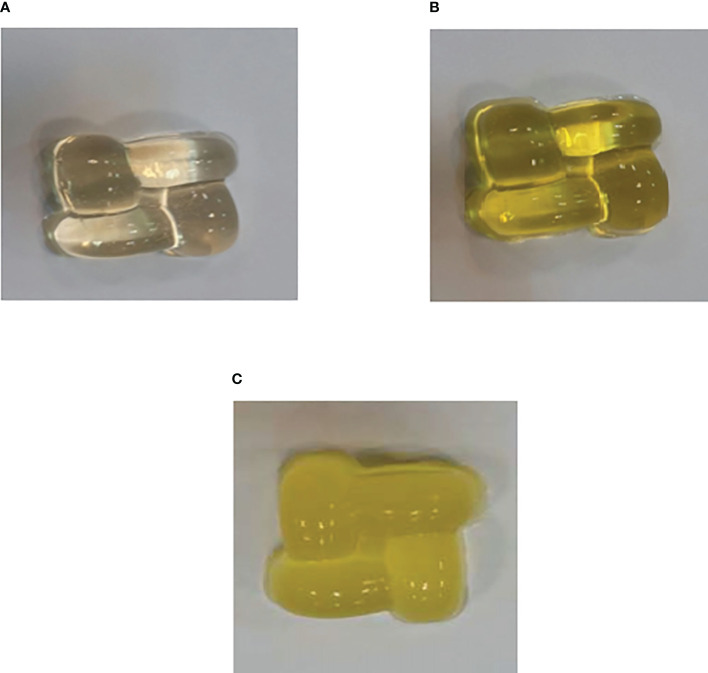
Gummy candies were produced for the study. **(A)** Only gummy candy, **(B)** Free BER in gummy candy, and **(C)** BER-loaded liposomes in gummy candy. 5 and 7.4 after 80 h.

Three types of gummy candy were prepared for further investigation, including only gummy candy, free BER in gummy candy, and BER-loaded liposomes in gummy candy (**Figure 5**).

#### 3.3.1 Sensory analysis

Sensory analysis of food utilizes the senses of smell, taste, sight, hearing, and touch. As mentioned above, sensory evaluation was carried out by recording the responses of assessors (ten random consumers) after testing a product (30). In this study, the sensory test was based on the seven-point hedonic scale to reflect the preferences of consumers. The evaluation results of Free BER in gummy candy showed that it was declared very well in terms of color, relatively good in terms of texture, relatively good in terms of taste, and overall acceptance. The evaluation results of BER-loaded liposomes in gummy candy showed that it was declared relatively good in terms of color, neither good nor bad in terms of texture, relatively good in terms of taste and neither good nor bad in terms of overall acceptance. The obtained results indicated no considerable changes in the taste and color of gummy candies containing the free BER and the BER-loaded liposome.

#### 3.3.2 Texture analysis of gummy candies

Texture profile analysis (TPA) involves two cycles of compression force, which can be applied to measure the compression test on functional gummies. In this work, TPA was used to study the effects of free BER and BER-loaded liposome on the mechanical properties of the prepared gummy candies, and the following parameters were recorded: hardness, cohesiveness, springiness, and chewiness, which indicate the firmness, the stickiness, the elasticity, and the tenderness of the prepared gummy candies, respectively (31, 32). The instrumental texture measurements of the gummy candies containing free BER and BER-loaded liposomes were calculated and the results are shown in **Table 1**. As shown in this table, gummy candies containing BER-loaded liposomes had high stickiness, more elastic and tenderness, but with lower strength compared to the gummy candies containing free BER and the control sample (6, 25, 33).

**Table 1:** Some physicochemical properties of the designed gummy candies.

Sample	Hardness (g)	Cohesiveness	Gumminess (g)	Chewiness (gmm)	Springness (mm)	Water activity
**GC**	1259 ± 4	0.91 ± 0.00	1160 ± 8	6830 ± 110	5 ± 0.01	0.54 ± 0.01
**GBL**	1321 ± 213	0.91 ± 0.01	731 ± 218	8921 ± 1602	4 ± o.2	0.61 ± 0.01
**GBF**	1421 ± 213	0.92 ± 0.03	928 ± 191	7812 ± 341	5 ± 0.01	0.59 ± 0.03

GC, gummy candy; GBL, BER-loaded liposomes in gummy candy; GBF, Free BER in gummy candy.

#### 3.3.3 Assessment of water activity

The a_w_ of food is the ratio between the vapor pressure of the food itself and the vapor pressure of distilled water under identical conditions. The a_w_ of the gummy candies is shown in **Table 1**. According to this table, the a_w_ of the gummy candies containing free BER and BER-loaded liposomes showed no significant differences from the a_w_ of the control sample. However, the prepared gummy candies comply with the range of a_w_ recommended for this type of product (0.54 to 0.66) (25).

### 3.4 The results of berberine revitalization activity

#### 3.4.1 DPPH radical scavenging activity

BER is a natural compound with high antioxidant and radical scavenging activities. DPPH radicals form a violet-colored solution when dissolved in ethanol. In this work, the antioxidant activity of free BER and BER-loaded liposomes in gummy candy formulations was tested using DPPH and ABTS methods for 60 days. In the presence of antioxidant molecules, these free radicals are reduced, and the color of the reaction mixture changes from purple to yellow, with a decrease in absorbance at 517 nm. The obtained results are shown in **Table 2**. It can be seen from this table, that during 60 days, the percentage of radical inhibition activity of gummy candies containing BER-loaded liposomes and free BER changed from 84.5% to 80.5% and 92.5 to 68.5%, respectively. Standard deviation in DPPH assay for BER in 0, 15, 30, 45, and 60 days were 0.707, 0.707, 1.414, 1.414, 0.707 and for BER-liposomal in 0, 15, 30, 45, and 60 days were 2.121, 0.707, 1.414, 0.707, 0.707 respectively (p<0.05). Liposomes containing BER hydrochloride have been studied for their antioxidant properties. These liposomes were prepared using the thin layer dispersion hydration method and as a liposome-gel. In the study by Shen et al. (34), the results showed that the entrapment efficiency of BBH-L, prepared by the thin layer dispersion hydration method, was 78.56 ± 0.7% with a particle size of 155.4 ± 9.3 nm. BBH-L-Gel demonstrated good scavenging capacity for DPPH and H2O2 and effectively inhibited the production of hepatic lipid peroxide MDA in the concentration range of 0.4-2.0 mg/mL. Additionally, the study by Wang et al. (35) indicated that liposome-gel loaded with BER hydrochloride exhibited strong antioxidant properties, as evidenced by its ability to inhibit DPPH and H2O2 free radicals and resist lipid peroxidation.

**Table 2:** DPPH and ABTS radical-scavenging activity of free BER and BER-loaded liposomes.

Revitalization activity	Gummy candies Free BER/BER-loaded liposome	Time(days)					P value
		0	15	30	45	60	
***DPPH***	BER (%)	92.5 ± 0.5	80.5 ± 0.5	75 ± 1.0	72 ± 1.0	68.5 ± 0.5	**p<0.05**
	SD	0.707	0.707	1.414	1.414	0.707	
	BER-loaded liposome (%)	84.5 ± 1.5	89.5 ± 0.5	87 ± 1.0	84.5 ± 0.5	80.5 ± 0.5	**p<0.05**
	SD	2.121	0.707	1.414	0.707	0.707	
***ABTS***	BER (%)	97.5 ± 0.5	82.0 ± 0.5	78.5 ± 0.5	72.5 ± 1.5	70.0 ± 2.0	**p<0.05**
	SD	0.707	0.707	0.707	2.121	2.828	
	BER-loaded liposome (%)	89.0 ± 0.5	93.0 ± 1.0	89.5 ± 0.5	88.0 ± 1.0	82.5 ± 0.5	**p<0.05**
	SD	0.707	1.414	0.707	1.414	0.707	

DPPH, Potassium persulfate, 1, 1-diphenyl-2-picrylhydrazyl; BER, Berberine; ABTS, 2, 2′-azinobis 3-ethylbenzothiazoline-6-sulphonic acid; SD, standard deviation.

Bold means that P value < 0.05 was considered statistically significant.

#### 3.4.2 ABTS radical scavenger activity

To investigate the radical scavenging activity of prepared gummy candies, ABTS assay was used. The principle of ABTS assay is based on the reduction of the blue-green ABTS radical by hydrogen-donating antioxidants and decreasing absorbance at wavelength 734 nm (36). As shown in **Table 2**, the absorption of active ABTS solution decreased from 97.5% to 70% and from 89.55% to 82.5% in the presence of gummy candy formulations containing BER-loaded liposomes and free BER over 60 days (**Table 2**). So, in agreement with the previous method, the radical inhibition activity of gummy candies containing BER-loaded liposomes was higher than that of free BER. Therefore, it can be concluded that nano-liposome encapsulation of BER can preserve the antioxidant activity of BER from adverse environmental conditions. It has been reported that encapsulation protects the core material from adverse environmental conditions, such as undesirable effects of light, moisture, and oxygen, thereby contributing to an increase in the shelf life of the product, and promoting a controlled liberation of the encapsulate (37, 38).

### 3.6 *In vitro* cytotoxicity studies

**Figure 6:**
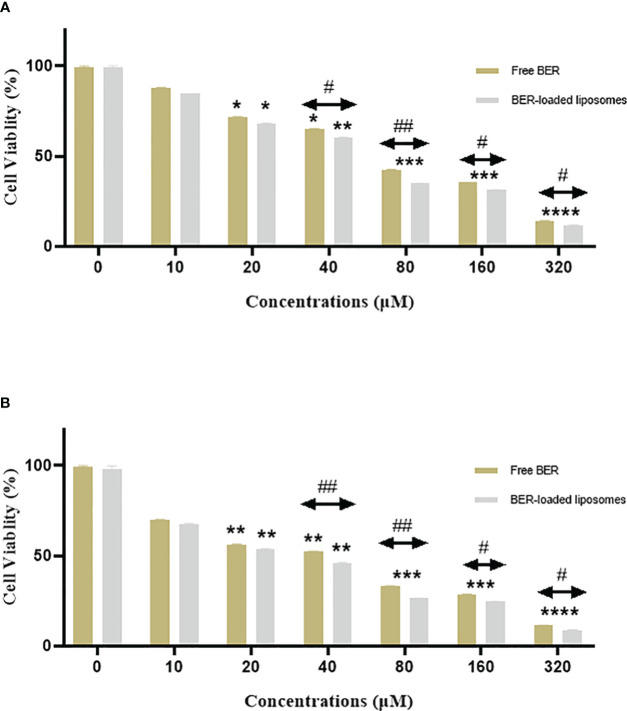
This chart compares the viability of the MKN-45 cells treated with free BER and BER-loaded liposomes after **(A)** 24 and **(B)** 48 hours. *P < 0.05, **P < 0.01, ***P < 0.001, and ****P < 0.0001. Indicate significant differences between the treatment groups and the control. #P < 0.05, and ##P < 0.01. Show the significant difference between free BER and BER-loaded liposomes.

The antiproliferative effect of free BER, and BER-loaded liposome was evaluated on the MKN-45 gastric cancer cell line by the MTT assay. For this purpose, 10,000 cells/well were treated with different concentrations of free BER, and BER-loaded liposomes for 24, and 48 h. The results indicated the cytotoxic effect of the studied compounds on the MKN-45 gastric cancer cell line. The IC50 value of free BER (66.72 μM) and BER-loaded liposome (52.58 μM) on MKN-45 cancer cells was after 24 h (**Figure 6A**). The IC50 value of free BER (36 μM) and BER-loaded liposome (28.63 μM) on MKN-45 cancer cells was after 48 h (**Figure 6B**).

The viability of the gastric cancer cells was decreased in the presence of BER and BER-loaded liposomes in a time and concentration-dependent manner. According to studies conducted by Liu Bing et al. (22), BER inhibits human hepatoma cell invasion without causing cytotoxicity in healthy hepatocytes. Pharmacokinetic studies conducted in 2011 have shown that BER is poorly absorbed in humans, making it difficult to maintain a 40 µM plasma concentration after oral administration. Consequently, BER is a dose-dependent compound: at physiological concentrations, it exhibits antioxidant properties, while at high concentrations, it induces cancer cell death.

### 3.7 Flow cytometric assessment of apoptosis on normal vascular endothelial cells

**Figure 7:**
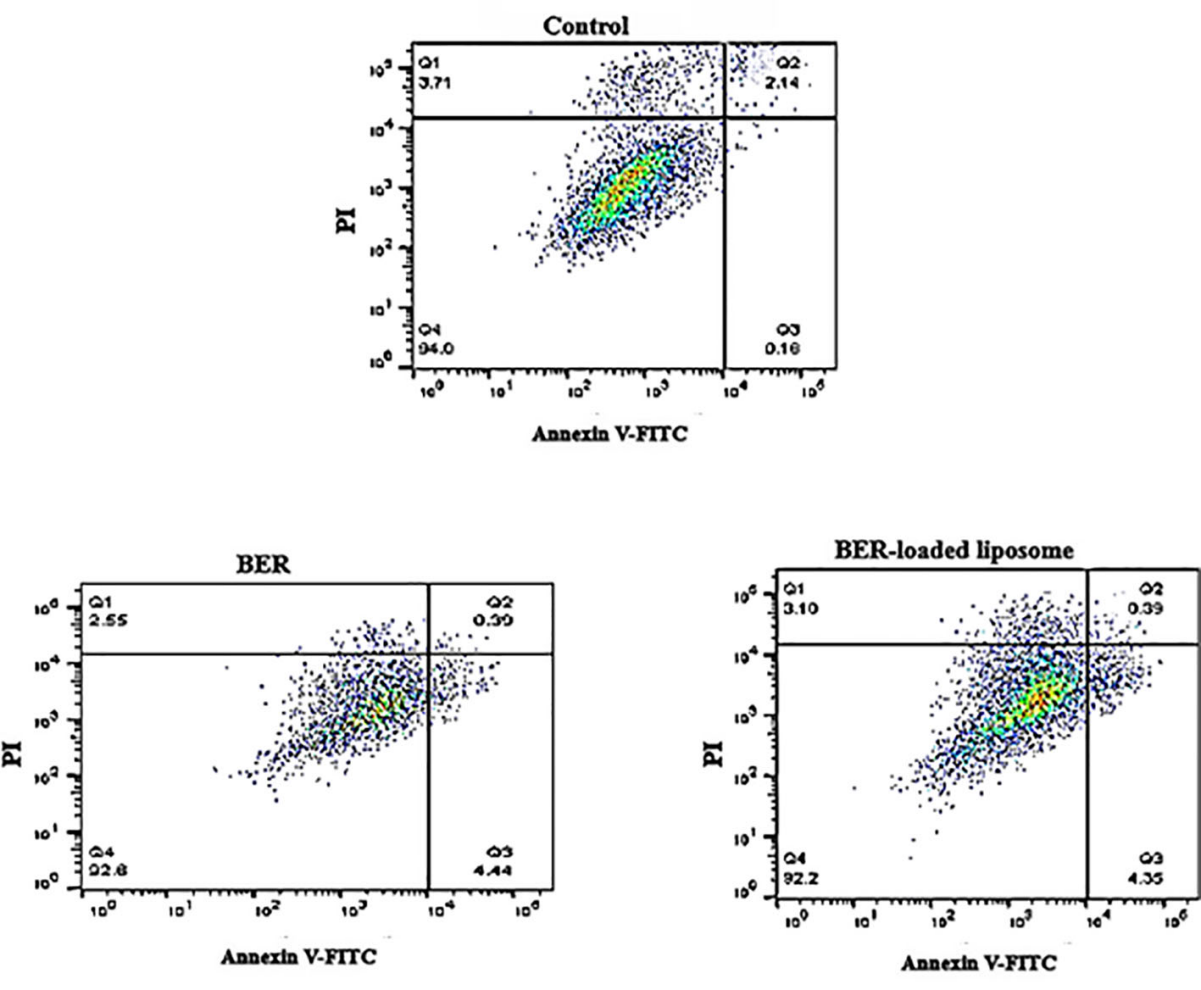
Investigating the effect of free BER and BER-loaded liposomes on apoptosis in normal vascular endothelial cells.

A flow cytometry technique was applied to investigate the apoptotic effects of free BER and BER-loaded liposomes on HUVEC cells. For this purpose, HUVEC cells were treated with free BER (66.72 μM) and BER-loaded liposome (52.58 μM) as apoptosis inducers. The results showed that BER, both in free form and in liposome form, causes the apoptosis of 0.39% of HUVEC cells (**Figure 7**). Most of the cells survived without entering the cell death phase. In a study by Liu Bing et al. (22), BER inhibited human hepatocyte invasion without causing cytotoxicity in healthy hepatocytes. It was found that BER has a suppressive effect on HepG2 cells without inducing cytotoxicity in normal liver cells.

### 3.8 Flow cytometric assessment of apoptosis on MKN-45 cells

**Figure 8:**
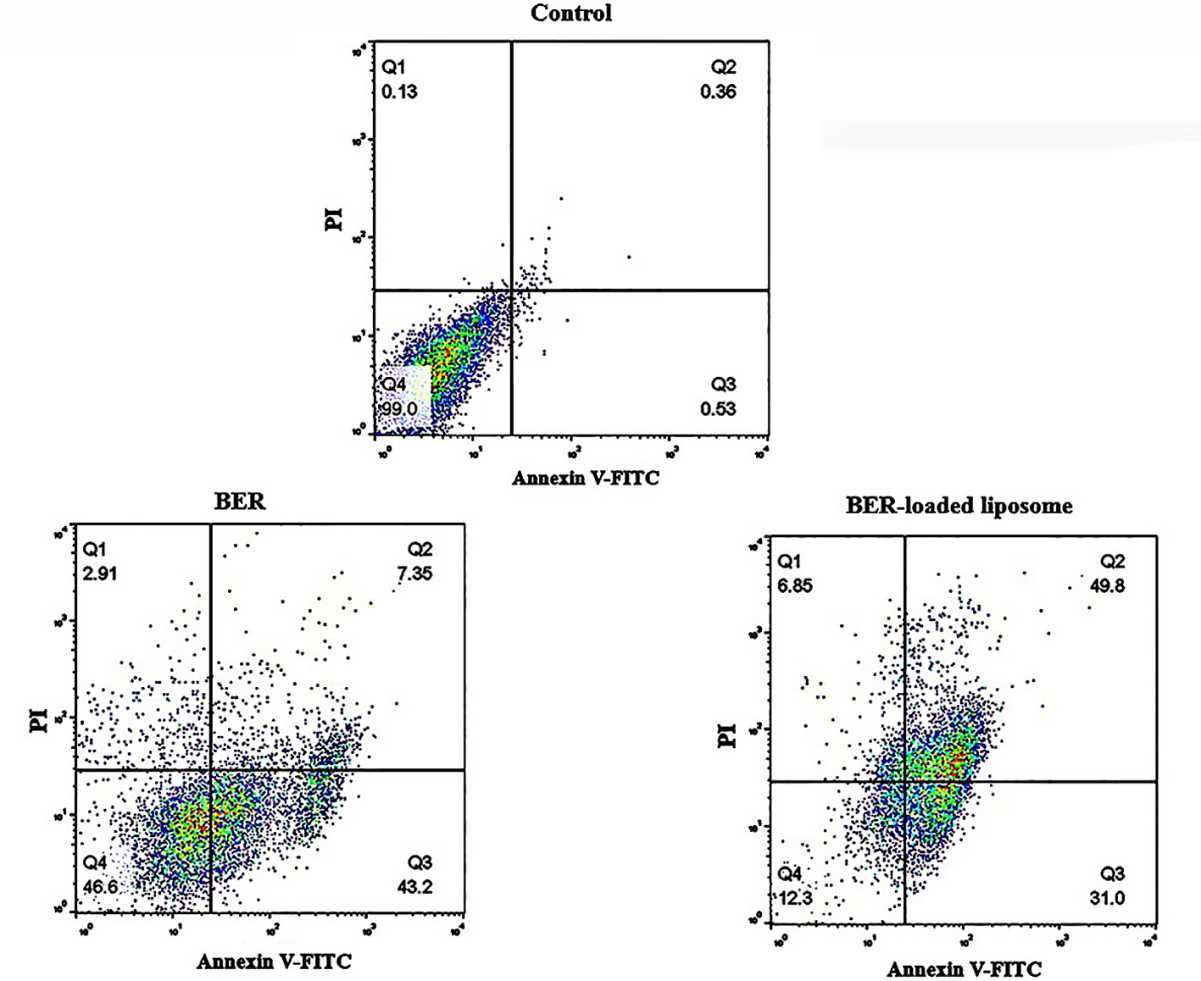
Effect of free BER and BER-loaded liposomes on induction of apoptosis in MKN-45 gastric cancer cell line.

A flow cytometry technique was applied in order to investigate the apoptotic effects of BER and BER-loaded liposomes on MKN-45 cells. For this purpose, MKN-45 cells were treated with free BER (66.72 μM) and BER-loaded liposome (52.58 μM) as apoptosis inducers. The flow cytometric method has been widely used to distinguish between intact cells (Annexin −/PI −), late apoptotic cells (Annexin +/PI +), early apoptotic cells (Annexin +/PI −), and necrotic cells (Annexin −/PI +) (39). Flow cytometry analysis of the MKN-45 cells treated with free BER and BER-loaded liposomes is shown in **Figure 8**. As shown in this figure, in comparison with the control cells, free BER and BER-loaded liposome can induce apoptosis and the treated MKN-45 cells shifted from early to late apoptosis or necrosis. However, the apoptotic effect of the BER-loaded liposome was higher than that of free BER. The data for early apoptosis, late apoptosis, and necrosis were calculated to be 43.2%, 7.35%, and 2.91% for MKN-45 cells treated with free BER, respectively. Also, the data for early apoptosis, late apoptosis, and necrosis were calculated to be 31%, 49.8%, and 6.85 for MKN-45 cells treated with BER-loaded liposomes, respectively. These results indicate that free BER (66.72 μM) and BER-loaded liposome (52.58 μM) are able to induce apoptosis in MKN-45 cell lines.

## 4 Conclusion

In conclusion, in the present work, BER-loaded nano-liposomes were successfully prepared by thin film hydration technique, and the poorly water-soluble BER was efficiently encapsulated into the nanoparticles for oral administration. The prepared formulation was characterized using different methods and the results confirmed liposomes with uniform size and relatively high active load (EE and DL values of 99% and 4.9%, respectively). The enriched gummy candies were prepared using free BER and BER-loaded liposomes. The physicochemical properties of the prepared gummy candies were analyzed and texture profile analysis gave valuable insights into the gummies’ mechanical properties, which are cohesiveness, springiness, hardness, gumminess, and chewiness. The antioxidant activity of the gummy candies containing free BER and BER-loaded liposomes was evaluated by ABTS and DPPH tests. The results showed that gummy candy formulations containing BER-loaded liposomes maintain their antioxidant potential even after 60-day incubation (compared with free BER).

On the other hand, the antiproliferative effect of free BER, and BER-loaded liposomes was evaluated on the MKN-45 gastric cancer cell line. The results indicated the cytotoxic effect of the studied compounds on the MKN-45 gastric cancer cell line. MTT assay results indicated that BER-loaded liposomes were more toxic to the MKN-45 cell line than free BER. Flow cytometric data suggested the high anticancer effect of BER-loaded liposomes (compared with free BER). These results indicated that encapsulation of BER protects its antioxidant activity and increases its bioavailability. Also, encapsulation can promote a controlled liberation of the BER, which makes it favorable for oral administration in the form of gummy candies.

To further substantiate these findings, it is recommended that future studies include animal experiments to evaluate the *in vivo* efficacy and safety of BER-loaded nano-liposomes. Additionally, the anticancer activity of BER-loaded nano-liposomes should be tested on other gastrointestinal cancer cell lines. Conducting these studies will provide relevant research data to support the potential therapeutic use of BER-loaded liposomes in gastrointestinal cancers and other related conditions.

## Data Availability

The raw data supporting the conclusions of this article will be made available by the authors, without undue reservation.
